# Shaping cancer center priorities through Community Advisory Board collaboration

**DOI:** 10.1186/s40900-025-00690-7

**Published:** 2025-03-10

**Authors:** Stephanie Evett, Megan E. Schmidt, Sarah H. Nash, Kelly Wells Sittig, University of Iowa Health Care Holden Comprehensive Cancer Center Community Advisory Board, Natoshia M. Askelson

**Affiliations:** 1 University of Iowa College of Public Health, 145 N. Riverside Dr, Iowa City, IA 52242 USA; 2 University of Iowa Health Care Holden Comprehensive Cancer Center, Iowa City, IA USA; 3 State Health Registry of Iowa, Department of Epidemiology, College of Public Health, University of Iowa, Iowa City, IA USA; 4 Iowa Cancer Consortium, Iowa City, IA USA

**Keywords:** Community outreach and engagement, Community Advisory Board, Strategic planning, Priorities, Cancer

## Abstract

**Background:**

Community Outreach and Engagement (COE) teams at National Cancer Institute-designated comprehensive cancer centers are tasked with engaging communities to understand and address the burden of cancer within their catchment area. This helps cancer center leadership identify priorities and develop strategic plans to reduce the cancer burden. University of Iowa Health Care Holden Comprehensive Cancer Center collaborates with its Community Advisory Board (CAB) to understand the needs and priorities of its catchment area, the state of Iowa.

**Methods:**

The Holden Comprehensive Cancer Center’s CAB is made up of diverse individuals from across the state, including community leaders and representatives from local hospitals, health departments, and nonprofits, who are dedicated to partnering with Holden Cancer Center to reduce the state's cancer burden. Holden Cancer Center’s COE team engaged its CAB in conversations to establish a process to identify research, clinical, and outreach priorities for the Holden Cancer Center. Small- and whole-group dialogues during CAB meetings helped gauge important criteria for determining priorities. The COE team also conducted online surveys to quantitatively assess CAB perspectives on guiding criteria for Holden Cancer Center to consider when identifying priorities.

**Results:**

Over the course of three interactive meetings, CAB members refined and ranked criteria for selecting Holden Cancer Center priorities. The top guiding criteria identified by CAB members included barriers patients face to cancer treatment, screening, or clinical trials; preventable cancers; and achieving health equity or correcting health disparities in the state.

**Conclusion:**

The COE team, Holden Cancer Center leadership, and CAB members are exploring avenues to inform Holden Cancer Center research and program planning with priorities developed using the CAB-identified guiding criteria. The multi-step process employed by Holden Cancer Center to engage community members in the identification and creation of priorities can be adapted by other cancer centers to ensure that institutional and community priorities are aligned.

**Supplementary Information:**

The online version contains supplementary material available at 10.1186/s40900-025-00690-7.

## Abstract (plain language summary)

The Community Outreach and Engagement (COE) team at University of Iowa Health Care Holden Comprehensive Cancer Center collaborated with its Community Advisory Board (CAB) to determine research priorities for the Holden Cancer Center to reduce the burden of cancer in the state of Iowa. To determine priorities, the CAB participated in small- and whole-group conversations, and took online surveys to decide what was most important for Holden Cancer Center to consider. CAB members felt the top priorities for Holden Cancer Center should be barriers patients face to cancer screening or treatment; cancers that can be prevented; and health equity. As the CAB represents the communities served by Holden Cancer Center, involving the CAB in this process ensured the priorities of the cancer center aligned with the needs of the community. Other cancer centers can follow a similar process with their CABs to develop priorities aimed at reducing the burden of cancer in their communities.

**Supplementary Information:**

The online version contains supplementary material available at 10.1186/s40900-025-00690-7.

## Background

A National Cancer Institute (NCI)-designated comprehensive cancer center’s Community Outreach and Engagement (COE) team is tasked with engaging communities to understand and address the burden of cancer within their catchment area, the self-defined geographic area that a cancer center intends to serve [1, 2]. This assists cancer center leadership in identifying priorities and developing strategic plans to reduce the cancer burden in the catchment areas [1, 3]. Working with communities in the catchment area should involve bidirectional conversations with community representatives, including but not limited to, regular meetings and interactions with a Community Advisory Board (CAB) [1]. The CAB is an essential avenue for a cancer center to understand the needs and priorities of its catchment area and facilitates bidirectional engagement between the cancer center and the communities it serves [1].

Interaction with the cancer center’s CAB is crucial to ensure the priorities of the center align with the community’s needs and interests; without this interaction, the cancer center will be less effective at impacting cancer incidence rates. Cancer centers have engaged their CAB in strategic planning to help determine priorities for the cancer center, such as brainstorming sessions and surveys [4] and a thorough cancer needs assessment process [5, 6]. In conjunction with the CAB at University of Iowa (UI) Health Care Holden Comprehensive Cancer Center, the COE team employed a multi-step process to identify guiding criteria for Holden Cancer Center to consider when determining priorities (e.g., priority cancers, risk factors) to reduce the burden of cancer in the catchment area. We know that CABs are critical to the effective work of cancer centers [1]; therefore, here we describe our multi-step process to identify and direct research priorities, which may be a useful model for other cancer centers.

## Methods

### Study setting

UI Health Care Holden Comprehensive Cancer Center claims the state of Iowa as its catchment area. Nearly 40% of Iowa’s 3.2 million residents live in a rural county [7]. Most Iowans identify as White, at 89.8%, while 4.4% identify as African American or Black, 0.6% identify as American Indian or Alaska Native, 2.8% identify as Asian, 0.2% identify as Native Hawaiian or Other Pacific Islander, and 2.2% identify as two or more races [8]. Nearly 7% of Iowans identify as Hispanic or Latino [8]. Just over 22% of Iowans are under the age of 18, and nearly 20% are over the age of 65 [8]. Among all states in the US, Iowa has the second highest cancer incidence, with an estimated 21,000 new cancers diagnosed in 2024 [9]. Iowa also has the fastest growing cancer incidence rate in the US [9], and is the only state with a consistently increasing age-adjusted incidence rate over the past 3 years [10].

Formed in 2019, the Holden Comprehensive Cancer Center CAB comprises Iowans with diverse backgrounds and experiences, all of whom have an interest in reducing the burden of cancer in the state. The CAB was initially created in partnership with the Iowa Cancer Consortium, Iowa’s comprehensive cancer control coalition, to identify and recruit members for the CAB. CAB members serve 2-year terms, with the opportunity to renew their term once. CAB and COE team members frequently assess what communities are un-/underrepresented on the board, and a formal request for nominations goes out to CAB members each spring. The CAB comprises approximately 20 thought leaders and Iowa-based partners, including public health and non-profit workers, cancer survivors, and representatives from community cancer centers and clinics. Demographic and other relevant information on the CAB involved in this process can be found in Table 1. The CAB meets quarterly; activities include members providing feedback to researchers, reviewing grant proposals, participating in key Holden Cancer Center activities such as the annual research retreat, and informing strategic planning. Additionally, Iowa Cancer Registry data is regularly shared and discussed during CAB meetings. As the cancer center prepared to identify priorities of its most recent strategic plan, CAB members were asked to identify criteria Holden Cancer Center should consider when determining research, clinical, and outreach priorities. The ultimate goal of the process was not to achieve consensus, but rather to determine what CAB members felt was most important to consider after ongoing discussions.

**Table 1:** Demographics of CAB members

	FY 2023	FY 2024
*Gender*		
Male	3	3
Female	16	17
Race/Ethnicity*		
White	13	12
Black/African American	3	5
Hispanic/Latino	1	0
Native American/American Indian	2	2
Asian/Pacific Islander	0	0
*Age*		
18–34	1	2
35–50	10	10
51–64	7	7
65+	1	1
*Rurality*		
Rural County	5	8
Urban County	14	12
Organization Type**		
Non-Profit	10	10
Public Health	3	3
Health Care	3	6
Government	0	1
Education	3	1
Not affiliated with an organization	1	1

*Not all CAB members indicated their race/ethnicity

**Some CAB members had multiple organizations

### Meeting 1: discussion of cancer-relevant criteria

Prior to virtual meeting 1, the COE team brainstormed an initial list of cancer-relevant criteria, including incidence rates, mortality rates, survival rates, state and/or national priorities, and disparities, considering their different perspectives and areas of expertise, as well as the current social-political climate in Iowa. The initial list contained definitions and resources to facilitate a shared understanding of the criteria and was sent to CAB members prior to meeting 1 (Table 2). This was not intended to be an exhaustive list but serve as a starting point to begin discussions about determining priorities with the CAB. Fourteen CAB members completed a pre-meeting online survey ranking the initial criteria from most to least important, adding their own criteria to the survey if they felt any were missing. During meeting 1, a member of the COE team shared the kinds of data (e.g., incidence and mortality rates) that could be used to inform the development of criteria. CAB members were then placed in Zoom breakout rooms to discuss criteria and their individual survey responses. Following the breakout sessions, summaries of the small-group conversations were shared with the whole group. At the end of meeting 1, CAB members completed a second survey that asked them to indicate the top three criteria they felt were most important for consideration. The number of times each criterion was selected in the top three were counted.

**Table 2:** Initial list of criteria shared with CAB members

Criteria	Definition & additional resources
Incidence rates	The number of new cases of cancer per 100,000 each year. If interested, you can find incidence rates for Iowa from CDC & NIH and also from the Iowa Cancer Registry.
Mortality rates	The number of cancer deaths per 100,000 each year. If interested, you can find mortality rates for Iowa from CDC & NIH and also from the Iowa Cancer Registry
Cancer survivors	The number of people who currently have or have had cancer. The 2022 Cancer in Iowa report shares information on the number of cancer survivors in Iowa (page 5)
Early-onset	Cancer that is diagnosed at a younger age than would be expected (i.e., breast cancer diagnosed under age 45, colon cancer diagnosed under age 50)
Morbidities/health complications	Some cancers and cancer treatments lead to a higher number of other health related complications than other cancers
Economic impact	Some cancers have a greater economic impact on patients and/or the health care system than others
Screening rates	Some cancers have screening tests to help detect cancer at earlier stages. The 2022 Cancer in Iowa report provides information on the percentage of Iowans being screened for various types of cancer
Greater burden in Iowa	Some cancers have a greater burden in Iowa than they do in surrounding states. Iowa has among the highest rates of leukemia in the country, and the 5th highest rate of non-Hodgkin lymphoma
Disparities	Some groups bear a disproportionate burden of cancer compared to other groups, and the differences may be greater or less depending on the cancer. Disparities can be seen in incidence rates, mortality rates, screening rates, and stage at diagnosis, to name a few. The 2021 Cancer in Iowa report focused on some of the racial/ethnic cancer disparities in our state. Some examples of where we might see disparities include rural vs urban communities, in racial/ethnic groups, in insured vs uninsured, and by sexual orientation/gender identity
Curability or treatability	Some cancers are harder to cure or treat than others
Prognosis	Some cancers have a more complicated prognosis and/or a shorter survival time than other cancers
Alignment with community, state, and/or national priorities	Some community, state, and/or national partners create priority areas. These partners may include organizations like the American Cancer Society, Healthy People 2030, Iowa Department of Health and Human Services Cancer Programs & Division of Tobacco Control, Healthy Iowans, Cancer Moonshot, American Association for Cancer Research, etc.
Gaps in data/evidence	What can’t existing research and data tell us, and how can we build evidence in those areas? Who or what isn’t represented in existing research and how can we design research to address those gaps?
Alignment with the Iowa Cancer Plan	The Iowa Cancer Plan provides direction for planning, implementing, and evaluating cancer control programs, research, and policy initiatives

### Meeting 2: development of guiding questions

During virtual meeting 2, the COE team reviewed why Holden Cancer Center needs to determine priorities, shared examples to illustrate how priorities may be determined, and provided a summary of responses CAB members shared during the initial discussion in meeting 1. Keeping in mind the criteria generated and discussed in meeting 1, the 15 CAB members who attended meeting 2 were then placed into Zoom breakout rooms to create 3–5 guiding questions and/or criteria Holden Cancer Center should ask to determine if something is a priority for the catchment area. A member from each breakout room shared the generated questions and criteria with the whole group. The COE team synthesized the guiding questions and criteria generated by CAB members into a list of questions prior to meeting 3. We intentionally chose to pose the final criteria as questions since questions evoke consideration and reflection, which we felt better fit the nature of the activities the questions will be used for.

### Meeting 3: synthesis and ranking of guiding questions

In virtual meeting 3, the COE team shared the synthesized questions with CAB members. CAB members were asked to confirm that the synthesis was an accurate representation of the questions and criteria they created. At the end of the meeting, CAB members were sent a survey which asked them to rank the questions from most to least important. CAB members were informed that no questions would be eliminated, but that by ranking, Holden Cancer Center leadership and researchers could better understand what is especially important to the CAB and community. The survey was sent to all 20 CAB members, and one follow-up attempt was made via email with members who had not yet completed the survey. The scores for each guiding question were averaged and ranked in order of lowest to highest average.

## Results

### Meeting 1: discussion of cancer-relevant criteria

Fourteen out of 19 CAB members took the pre-meeting survey indicating what criteria they felt were most important to consider when determining priorities. Survey results can be found in Table 3. Results indicated CAB members felt cancer disparities, screening rates, and cancers with a greater burden in Iowa were the most important criteria to consider when establishing priorities.

**Table 3:** Meeting 1: Criteria CAB members considered as part of the priority determining process

Potential criteria brainstormed by COE team	CAB ranking before meeting 1 (n = 14)	CAB identified top 3 criteria after meeting 1 (n = 7)
Incidence rates	Disparities (m = 3.00, SD = 3.26)^a^	Screening rates (n = 5)^b^
Mortality rates	Screening rates (m = 3.78, SD = 2.81)	Disparities (n = 4)
Cancer survivors	Incidence rates (m = 5.64, SD = 3.56)	Curability or treatability (n = 3)
Early onset	Early onset (m = 5.92, SD = 2.59)	Gaps in data/evidence (n = 2)
Morbidities/health complications	Gaps in data/evidence (m = 6.00, SD = 4.71)	Alignment with the Iowa Cancer Plan (n = 2)
Economic impact	Mortality rates (m = 6.57, SD = 3.15)	Incidence rates (n = 1)
Screening rates	Greater burden in Iowa (m = 7.36, SD = 4.05)	Mortality rates (n = 1)
Greater burden in Iowa	Cancer survivors (m = 8.00, SD = 2.57)	Early onset (n = 1)
Disparities	Morbidities/health complications (m = 9.14, SD = 2.60)	Prognosis (n = 1)
Curability or treatability	Alignment with the Iowa Cancer Plan (m = 9.42, SD = 4.91)	Other—prevention (n = 1)
Prognosis	Curability or treatability (m = 9.78, SD = 2.94)	Cancer survivors (n = 0)
Alignment with community, state, and/or national priorities	Prognosis (m = 10.14, SD = 3.37)	Morbidities/health complications (n = 0)
Gaps in data/evidence	Economic impact (m = 10.14, SD = 2.41)	Economic impact (n = 0)
Alignment with the Iowa Cancer Plan	Alignment with community, state and/or national priorities (m = 10.92, SD = 3.34)	Greater burden in Iowa (n = 0)
Other (please specify)	Other—stage at diagnosis (m = 14.14, SD = 3.21)	Alignment with community, state, and/or national priorities (n = 0)

^a^Numbers in parentheses in this column indicate the mean rank, with lower means indicating CAB members viewed the criteria as more important, followed by standard deviation

^b^Numbers in parentheses in this column indicate the number of CAB members that chose this criteria as a top 3 criteria

Breakout room discussions focused on the unique opportunity Holden Cancer Center has in working on issues that do not receive much attention, highlighting the opportunity the cancer center has to fill these gaps. CAB members felt there were many important criteria to consider, that there was a lot of overlap between criteria, and that the criteria chosen should be data driven. This discussion indicated the top criteria to consider included prevention, data driven criteria, and a focus on incidence and mortality rates.

Seven out of 19 CAB members completed the post-meeting survey on the top three most important criteria to consider when establishing priorities. Survey results can be found in Table 3. These criteria included screening prevalence, cancer disparities and cancers with highly effective treatments (curability and treatability).

### Meeting 2: development of guiding questions

The complete list of questions and criteria generated by 15 CAB members during meeting 2 breakout rooms can be found in Table 4. Although there was variety in the questions and criteria generated by each breakout group, there were some common themes. Questions and criteria of common interest included those focused on health equity, patient barriers, and cancer data. CAB members had different perceptions as to which data were most important to consider, with one breakout room mentioning incidence and mortality, another mentioning use of population level data, and a third interested in comparing incidence rates over time. The importance of prevention and screening were also discussed. After the meeting, members from the COE team synthesized the proposed questions and criteria into a list of questions. The list of synthesized questions can be seen in Table 5.

**Table 4:** Meeting 2: Criteria CAB members discussed as part of the priority determining process

Breakout room 1 (n = 5)	Breakout room 2 (n = 5)	Breakout room 3 (n = 5)
Use population-level data to determine needs	Is this a leading cause in incidence or death in Iowa? What is the impact in Iowa?	What has changed? Why is Iowa #2?
Transportation: how can we help people get to appointments and screenings?	Does it address health equity or correct an imbalance?	What makes Iowa different? What makes our patient population different or comparable? Do some populations need to be focused in on gap care? Or language? Or race/ethnicity?
Screening	Can we catch it early and save lives?	What are the top barriers facing patients in Iowa for cancer treatment or screening?
Prevention	Is there community engagement? Can we get community support or advisory groups or social engagement to help?

**Table 5:** Meeting 3: Guiding questions CAB members considered as part of the priority determining process

Ranking	Mean rank (SD)	Guiding question
1	3.00 (2.54)	Do patients face significant barriers to cancer treatment, screening, or trials around this priority?
2	3.53 (1.85)	Is this priority a preventable cancer or related to a preventable cancer?
3	3.67 (1.99)	Does this priority address a health inequity or correct an imbalance in Iowa?
4	4.33 (2.66)	Is this priority area a leading cause of cancer incidence in Iowa?
5	5.27 (2.15)	Are there communities and partnerships that are ready to engage around this priority?
6	5.33 (2.02)	Is this priority area a leading cause of mortality in Iowa?
7	5.33 (2.19)	Is the incidence rate of this priority area rising in Iowa?
8	5.53 (1.77)	Is this priority a screenable cancer or related to a screenable cancer?

Each questions’ scores were averaged and ranked from lowest to highest (1 is most important, 8 is least important)

### Meeting 3: synthesis and ranking of guiding questions

The ranking results from the final survey completed by 15 CAB members after meeting 3 can be found in Table 5. The top 3 guiding questions CAB members felt Holden Cancer Center leadership should consider when setting priorities include: “Do patients face significant barriers to cancer treatment, screening, or trials around this priority?”, “Is this priority a preventable cancer or related to a preventable cancer?”, and “Does this priority address a health inequity or correct an imbalance in Iowa?”

## Discussion

Using a multi-step, iterative process, Holden Comprehensive Cancer Center’s CAB identified guiding criteria to help Holden Cancer Center develop priorities to reduce the burden of cancer in Iowa. Guiding questions included whether patients face barriers to cancer treatment, screening, or clinical trials; whether there are evidence-based prevention strategies known for the cancer; and whether we are achieving health equity or correcting health disparities in Iowa. These questions represent one key method that Holden Cancer Center has used to engage with community, through the CAB, to understand community priorities.

Engaging the CAB is a critical step when determining priorities for a comprehensive cancer center, as the CAB is an essential avenue for understanding the needs of the communities in the catchment area [1]. The process used by the Holden Comprehensive Cancer Center COE team expands the engagement described by previous cancer centers [4, 5]. For example, one comprehensive cancer center described a process wherein a range of participants, including their CAB, engaged in the center’s strategic planning process over the course of one year, involving surveys and brainstorming sessions to gather information and develop the strategic plan [4]. Another comprehensive cancer center involved its CAB in a strategic planning process consisting of a thorough cancer needs assessment that involved collecting data and concept mapping to identify priorities [5]. Similarly, a different cancer center engaged its CAB in a cancer needs assessment process, seeking their input on the goals of the assessment, data to be collected, and products to develop when disseminating findings [6]. Further, the extended process described here used different formats over time, and allowed our CAB members to participate in the process even if they were not present at every meeting.

One limitation of our work was the time it took to identify priorities. Involving a CAB in decision making requires substantial flexibility, adaptability, and time. This process was initially planned to take place during one or two CAB meetings; however, discussions took longer than anticipated. Additionally, approximately half of the CAB members had terms which ended midway through the process. Although it would have been preferable to complete the process before their terms ended, a summary of the initial discussions was shared with new members, which ultimately allowed for input from more CAB members. While the time intensive process of determining guiding criteria was a challenge, it also provided valuable insights that will inform future Holden Cancer Center priorities. Conducting this process in a real-world setting presented challenges, such as fluctuating attendance and variable participation rates in meetings and surveys. We anticipate this to be a common issue when working with advisory board and community members.

The Holden Comprehensive Cancer Center COE team is currently working with cancer center leadership and CAB members to integrate the guiding questions into different aspects of Holden Cancer Center’s operations. For example, Holden Cancer Center leadership used the questions in conjunction with Iowa Cancer Registry data to help identify the five current cancer center priorities: breast cancer, lung cancer, melanoma, prostate cancer, and survivorship. Additionally, the guiding questions will be considered as the cancer center finalizes its strategic plan. Other proposed avenues include considering the questions when selecting clinical trials to open, when applying for grants, and when engaging in faculty recruitment. Further, the guiding questions will prove useful in the context of grant funding; specifically, the guiding questions will be considered by cancer center leadership and grant reviewers when awarding internal funding. Researchers will be able to consider the guiding questions themselves when determining what grants to apply for and when writing grant applications. Additionally, the COE team has incorporated the guiding questions into their work. For example, the team is working to develop pilot funding for community partners; with this funding, grant applicants will be expected to explain how their proposed work ties into the guiding questions. Moreover, the COE team created an opportunity assessment tool to help identify, evaluate, and prioritize potential opportunities, and the CAB’s guiding questions were included as a key component of the tool. The COE team also has plans to hire an outreach worker based in rural Iowa to focus on implementing evidence-based interventions to address prevention, screening, survivorship, which aligns closely with several of the CAB’s guiding questions. These activities highlight that it is critical and possible not only to listen to community, but also to integrate what you learn into cancer center process and activities; without this, communication is not truly bidirectional, and the input of community is not truly engaged.

## Conclusion

Engaging the CAB is critical in ensuring the priorities developed by the cancer center align with the diverse needs of the community. This multi-step process to authentically engage community members in the identification and creation of priorities can be adapted by other cancer centers to ensure community involvement in the development of cancer center priorities to reduce the burden of cancer in the catchment area.

## Supplementary Information

Additional file 1.

## Data Availability

No datasets were generated or analysed during the current study.

## Abbreviations

CAB: Community Advisory Board
COE: Community Outreach and Engagement
NCI: National Cancer Institute
UI: University of Iowa
