# Supplementary material for: Shaping cancer center priorities through Community Advisory Board collaboration
Source: Res Involv Engagem. 2025 Mar 10;11:21. doi: 10.1186/s40900-025-00690-7 (PMC11895190; doi:10.1186/s40900-025-00690-7)
Supplement: Supplementary file 1 — Additional file 1. [file 40900_2025_690_MOESM1_ESM.docx]

GRIPP Checklist for Shaping Cancer Center Priorities through Community Advisory Board Collaboration Manuscript

GRIPP2 Short Form

| Section & Topic | Item | Reported on Page # |
| --- | --- | --- |
| 1: Aim | Report the aim of PPI in the study | 5 |
| 2: Methods | Provide a clear description of the methods used for PPI in the study | 6-9 |
| 3: Study Results | Outcomes- report the results of PPI in the study, including both positive and negative outcomes | 9-10 |
| 4: Discussion and conclusions | Outcomes- comment on the extent to which PPI influenced the study overall. Describe positive and negative effects | 10-13 |
| 5: Reflections/ critical perspective | Comment critically on the study, reflecting on things that went well, and those that did not, so others can learn from this experience | 11-13 |
